# Magnetic-Field-Assisted Assembly of Anisotropic Superstructures by Iron Oxide Nanoparticles and Their Enhanced Magnetism

**DOI:** 10.1186/s11671-016-1406-9

**Published:** 2016-04-12

**Authors:** Chengpeng Jiang, Chi Wah Leung, Philip W. T. Pong

**Affiliations:** Department of Electrical and Electronic Engineering, The University of Hong Kong, Hong Kong, Hong Kong; Department of Applied Physics, The Hong Kong Polytechnic University, Kowloon, Hong Kong

**Keywords:** Iron oxide nanoparticles, Nanoparticle superstructures, Nanoparticle assembly, Magnetic properties

## Abstract

Magnetic nanoparticle superstructures with controlled magnetic alignment and desired structural anisotropy hold promise for applications in data storage and energy storage. Assembly of monodisperse magnetic nanoparticles under a magnetic field could lead to highly ordered superstructures, providing distinctive magnetic properties. In this work, a low-cost fabrication technique was demonstrated to assemble sub-20-nm iron oxide nanoparticles into crystalline superstructures under an in-plane magnetic field. The gradient of the applied magnetic field contributes to the anisotropic formation of micron-sized superstructures. The magnitude of the applied magnetic field promotes the alignment of magnetic moments of the nanoparticles. The strong dipole-dipole interactions between the neighboring nanoparticles lead to a close-packed pattern as an energetically favorable configuration. Rod-shaped and spindle-shaped superstructures with uniform size and controlled spacing were obtained using spherical and polyhedral nanoparticles, respectively. The arrangement and alignment of the superstructures can be tuned by changing the experimental conditions. The two types of superstructures both show enhancement of coercivity and saturation magnetization along the applied field direction, which is presumably associated with the magnetic anisotropy and magnetic dipole interactions of the constituent nanoparticles and the increased shape anisotropy of the superstructures. Our results show that the magnetic-field-assisted assembly technique could be used for fabricating nanomaterial-based structures with controlled geometric dimensions and enhanced magnetic properties for magnetic and energy storage applications.

## Background

Enormous progress has been made in chemical synthesis of magnetic nanoparticles, enabling us to precisely control their morphology and composition [1–4]. To find their applications in data storage and energy storage, it is of fundamental and practical interest to organize these magnetic nanoparticles into structures with controlled shape, spacing, and alignment [4–7]. Self-assembly strategy by applying a magnetic field offers a simple and efficient route to assemble magnetic nanoparticles as the building blocks into well-ordered structures, providing distinctive magnetic properties related to coercivity and anisotropy energy [4, 7–9].

Generally, the magnetic-field-guided assembly of magnetic nanoparticles can be categorized into two types: dynamic self-assembly, being a nanoparticle organization process which occurs in liquid interfaces or solutions, with continuous energy input to grant the interactions responsible for the growing and sustaining of ordered structures; and static self-assembly, in which the nanoparticles assemble on solid surfaces or substrates, leading to stable structures or patterns without dissipating energy after the completion of assembly [10, 11]. A representative example of the liquid-phase assembly of magnetic nanoparticles is colloidal assembly of photonic crystals, and a number of research works have reported the dynamic assembly of magnetic colloidal crystals under applied field. Wang’s group demonstrated a magnetically actuated liquid crystal by controlling the assembly orientation and hence the transmittance intensity of magnetic nanorods using a weak magnetic field [12]. They further developed the magnetically responsive photonic structures built from magnetic nanoellipsoids, whose diffraction properties can be tuned reversibly [13]. Xia’s group introduced the colloidal clusters of magnetite nanocrystals into polymer matrix via photo-polymerization, and photonic bandgap of the resulting photonic crystals can be modulated by tuning the cluster size and field strength during self-assembly [14]. Timonen and his coworkers presented a switchable dynamic self-assembly technique by driving the ferrofluid droplets of iron oxide nanoparticles on super-hydrophobic surface under a time-vary magnetic field [15]. For the dynamic assembly of photonic crystals, manipulating the orientation or controlling the spacing of assembled structures is usually implemented by changing the applied magnetic field for the purpose of modulating optical properties [16, 17]. However, if magnetic storage application is envisioned, the assembled structures should be physically robust and mechanically stable without the presence of an external magnetic field. In this regard, magnetic-field-guided static assembly strategy is much preferred. Recent works on this topic mostly involve fabricating magnetic nanoparticle superstructures with alignment and shape anisotropy through evaporative or convective deposition process, during which geometric constraints or external stimuli could be applied to pattern or direct the assembled superstructures [18–21]. Park et al. achieved the production of crystalline superstructures in rod and wire shapes composed of 10-nm cobalt nanoparticles, which show enhanced coercivity associated with easy-axis anisotropy [22]. Li et al. demonstrated a solvent-evaporation assembly process to obtain micro-rod superstructures made of 21-nm magnetite nanoparticles, exhibiting specific crystallographic orientations [23]. Singh et al. developed an interfacial deposition method to assemble 13-nm magnetite nanocubes into aligned superstructures with stranded helical shapes [24]. Although periodic chain or rod structures in micron size with a high aspect ratio could be prepared in these reports and also in the recently developed strategies for static assembly of magnetic nanoparticles under a uniform magnetic field [22–26], fabrication of sub-10-μm nanoparticle superstructures with homogeneous morphology and controlled spacing still remains challenging. Besides, it will be of great value to investigate how the shape of the constituent nanoparticles and the gradient of the magnetic field influence the assembly process and measure the anisotropy-associated magnetic properties of the resulting superstructures. From the application viewpoint, the assembled superstructures with both shape anisotropy and magnetic anisotropy could be exploited as unique magnetic systems for studying nanomagnetism, and they could also be used for fabricating functional materials and solid-state magnetic devices with controlled magnetization direction and desired anisotropy energy.

In this work, sub-20-nm iron oxide nanoparticles (IONPs) with spherical and polyhedral shapes were synthesized by thermal pyrolysis in organic phase. An evaporative deposition method was applied to assemble the IONPs into superstructures under the applied magnetic field. The direction and rate of the assembly process and the magnitude and gradient of the magnetic field were controlled. Morphologies of the IONPs and their superstructures were characterized, and the assembly mechanism was analyzed. Effects of the experimental conditions and nanoparticle shape on the morphology and alignment of the superstructures were investigated. Magnetic properties of the superstructures were measured, and the enhancement of their coercivity and saturated magnetization along the applied field direction was investigated by calculating the magnetic anisotropy energy and dipole interaction energy of the IONPs.

## Methods

Iron (III) oxyhydroxide (99 %), iron (III) acetylacetonate (99.9 %), 1,2-tetradecanediol (90 %), oleic acid (90 %), lauric acid (98 %), and decanoic acid (98 %) were purchase from Aldrich. Octadecene (90 %) and benzyl ether (98 %) were obtained from Acros. All chemicals were used as received.

Spherical IONPs capped by lauric acid were synthesized using iron (III) oxyhydroxide (3 mmol), lauric acid (9 mmol), oleic acid (1 mmol), and octadecene (15 ml). The reaction mixture was vigorously stirred under N_2_, and it was first heated to 200 °C for 30 min and then heated to reflux for 1 h. By removing the heating source, the resulting black mixture was cooled to 50 °C and kept in a water bath. Excess acetone was added to the mixture, and a black-brown material was precipitated by centrifugation and then dissolved in hexane. This purification step was repeated two more times, and the obtained nanoparticles were dispersed in 6 ml of chloroform and toluene (*v*:*v* = 9:1).

Polyhedral IONPs capped by decanoic acid were synthesized using iron (III) acetylacetonate (3 mmol), decanoic acid (18 mmol), benzyl ether (60 ml), and 1,2-tetradecanediol (15 mmol). The heating process and the purification process were the same as those for spherical IONPs.

**Fig. 1:**
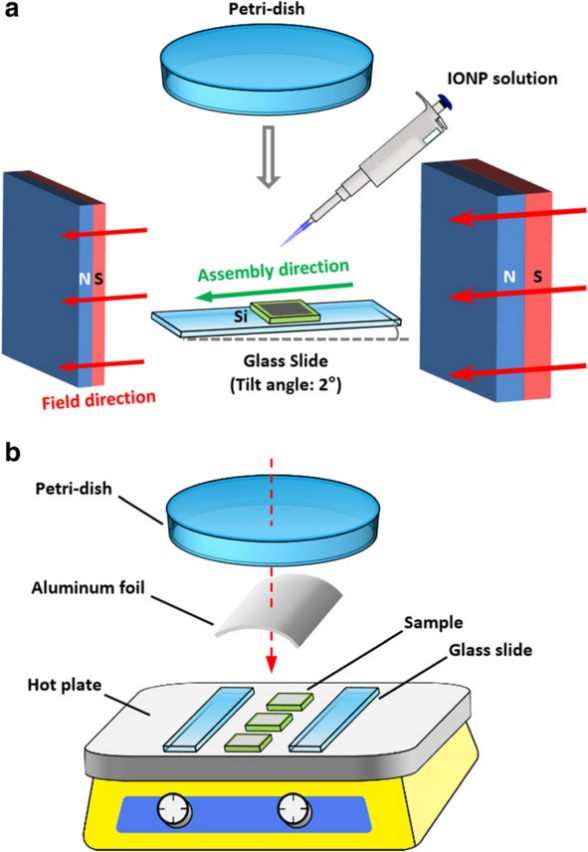
Schematic illustration of the experimental setups. **a** Magnetic-field-assisted assembly of the IONPs. **b** Heat treatment of the nanoparticle superstructures

Superstructures of the IONPs were prepared by depositing the diluted nanoparticle solution (volume 20 μl, concentration 160 mM based on Fe) onto a flat Si substrate (8 mm × 8 mm, thermally oxidized) subjected to an in-plane magnetic field generated by NdFeB magnets (Fig. 1a). The evaporation rate of the solvent was controlled by covering the sample with a petri dish, while the translation direction of the air-solution-substrate contact line was controlled by tilting the substrate at 1.5° with respect to the ground. The magnetic field direction was adjusted to be parallel to the translation direction of the three-phase contact line. The magnitude and gradient of the magnetic field (typical values at the initial assembly stage 200 Oe and 60 Oe/mm, measured at the sample center) were controlled by changing the distance and location of the magnets. After the solvent evaporated completely, the sample was transferred to a hot plate for annealing. Here, a hot plate was used instead of a convection oven due to that it heats the sample from the bottom by thermal conduction, which can prevent skin effect and shorten the heating time for thin film annealing. As shown in Fig. 1b, two glass slides were placed parallelly to fix the sample, and a curved aluminum foil and a glass petri dish were covered on top but without contacting the sample to prevent air flow and reduce heat dissipation. The sample was then heated at 200 °C for 15 min in air to remove the organic substances. After the heat treatment, a solid coating was obtained on the substrate surface.

The IONPs were characterized by transmission electron microscope (TEM). The nanoparticle superstructures were characterized using optical microscope and scanning electron microscope (SEM), and their magnetic properties were measured using vibrating sample magnetometer (VSM) and magnetic force microscope (MFM).

## Results and Discussion

**Fig. 2:**
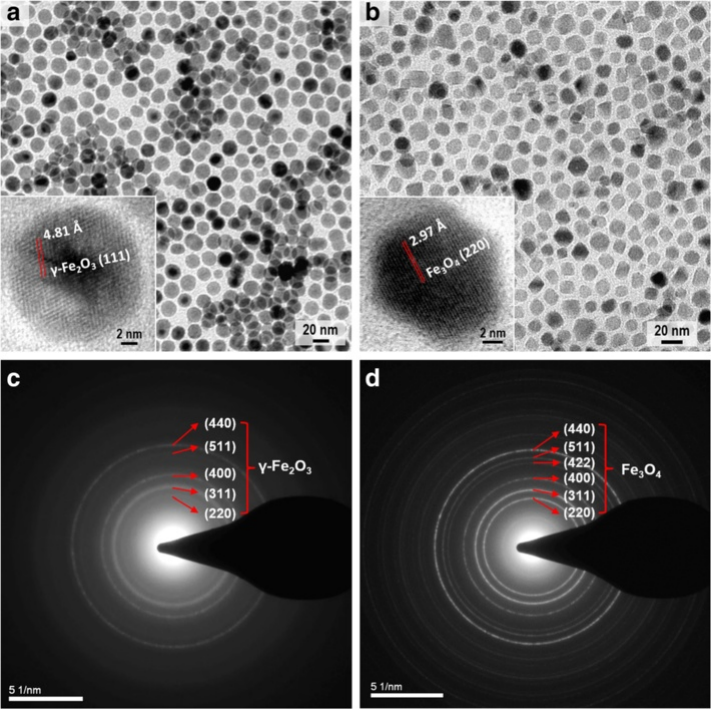
Characterization of the synthesized nanoparticles. **a** TEM image of spherical IONPs. **b** TEM image of polyhedral IONPs. *Inserts* show the HRTEM images. **c** SAED pattern of spherical IONPs. **d** SAED pattern of polyhedral IONPs

Morphology and composition of the synthesized nanoparticles were investigated by bright-field TEM, high-resolution TEM (HRTEM), and selected-area electron diffraction (SAED). Figure 2a shows that the lauric acid-capped nanoparticles are in spherical shape with an average size of 19 nm and their measured lattice spacing based on the diffraction rings in Fig. 2c matches well with the *d*-spacing of *hkl* planes in γ-Fe_2_O_3_ (JCPDF #04-0755). Figure 2b shows that the decanoic acid-capped nanoparticles are in polyhedral shape with an average size of 16 nm and their diffraction rings in Fig. 2d can be well indexed to the lattice spacing of Fe_3_O_4_ (JCPDF #19-0629). The TEM and HRTEM images demonstrate that the spherical nanoparticles are polycrystalline with a narrow size distribution of 8 %, while the polyhedral nanoparticles are single crystalline with a size distribution of 15 %. The ring features in both SAED patterns indicate the random orientations of the nanoparticles.

**Fig. 3:**
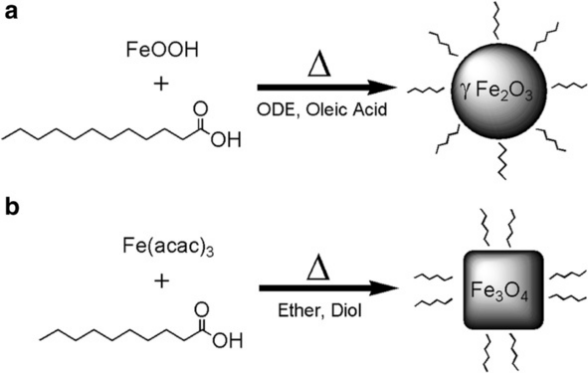
Schematic diagram. **a** Synthesis scheme of spherical IONPs. **b** Synthesis scheme of polyhedral IONPs

Synthesis protocols of the IONPs with different sizes and shapes are inspired by thermal pyrolysis procedures developed by William [27] and Sun [28] It was found that the chemical property of the precursor material and the coordinating ability of the organic solvent have great influence on the morphology of the IONPs. As illustrated in Fig. 3, the use of inorganic precursor and non-coordinating solvent led to isotropic growth of the IONPs with spherical shape, while using organometallic precursor and weakly coordinating solvent resulted in anisotropic growth of the IONPs with polyhedral shape. Surfactants with carboxylic groups were chosen as the capping agents for the IONPs due to their strong binding ability with the surface atoms of the IONPs, which contributes to the formation of nearly monodisperse nanoparticles with high yield and high stability. Decanoic acid and lauric acid were used instead of oleic acid since they have lower boiling point and shorter chain length, which could facilitate the removal of organic surfactant during heat treatment and also decrease the spacing between the neighboring IONPs for enhancing magnetic coupling. A temporal separation of the nucleation stage and the growth stage by kinetic and thermodynamic parameters was achieved using a two-step heating strategy, which helps to narrow the size distribution of the IONPs.

**Fig. 4:**
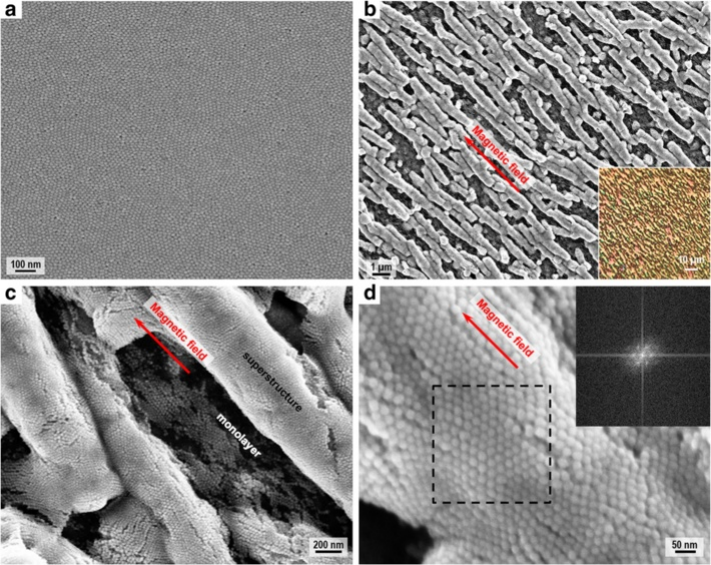
SEM images of the assembled structures prepared with spherical IONPs. **a** SEM image of the homogeneous film. **b** SEM image of the rod-shaped superstructures, with an *inset* of microscopic image. **c** Enlarged SEM image revealing the monolayer and superstructure. **d** Close-up SEM image and FFT pattern (*inset*) of the delineated region revealing the hexagonal packing of the nanoparticles

Morphology of the assembled structures formed by spherical IONPs is investigated by SEM. As shown in Fig. 4a, without the applied magnetic field, a homogeneous film composed of well-ordered spherical IONPs could be obtained. The nanoparticle film is compact and continuous without void or defect at micron scale, illustrating that the assembly technique by controlling the evaporation rate and assembly direction could lead to self-organized structures formed by monodisperse nanoparticles. As shown in Fig. 4b, under the in-plane applied magnetic field, rod-shaped superstructures with a length of 2.8 μm, width of 0.5 μm, and aspect ratio of ~6 were obtained, and they are homogeneously distributed with controlled spacing of 0.7 μm. By measuring the intersecting angle (*β*) between the applied magnetic field and the longitudinal axis of each rod-shaped superstructure, it is found that the superstructures show good alignment with the magnetic field (*β* = 4.0° ± 2.7°). The orientation of the superstructure may not accurately reflect the direction of the magnetic moments of its constituent IONPs, but it is obvious that the assembly directions of the superstructures correlate with the magnetic field direction. Besides, some dot-shaped superstructures were formed probably due to the incomplete growth of the assembled structures. Park et al. also reported the shape evolution of supercrystal dots during the magnetic assembly of supercrystal rods [22]. Enlarged SEM image in Fig. 4c reveals that the rod-shaped superstructures are composed of closely packed spherical IONPs without coalescence or crack and the dark regions between the adjacent superstructures are capped by a monolayer of IONPs. The ordered stacking structures in the close-up SEM image (Fig. 4d) and the six symmetric bright spots in the fast Fourier transform (FFT) pattern of the superstructure (inset of Fig. 4d) demonstrate that highly ordered hexagonal packing patterns were formed by uniform nanoparticles in the rod-shaped superstructures as an energetically favorable configuration [29].

**Fig. 5:**
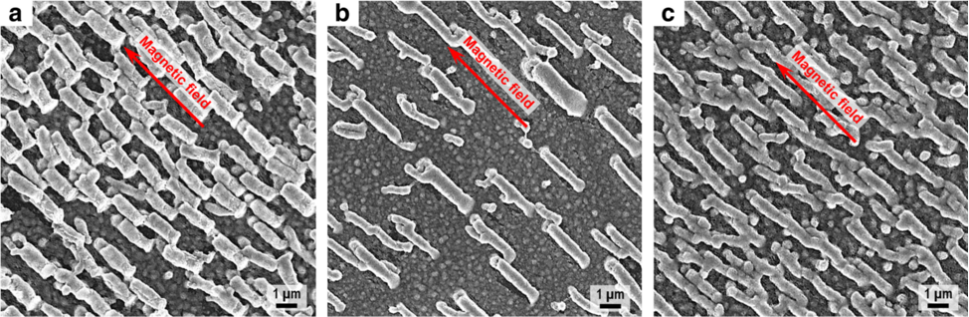
SEM images of the rod-shaped superstructures prepared under different conditions. **a** SEM image of the superstructures prepared by increasing the tilt angle of the substrate. **b** SEM image of the superstructures prepared by decreasing the concentration of the nanoparticle solution. **c** SEM image of the superstructures prepared by reducing the magnitude and gradient of the applied magnetic field

Effects of the experimental conditions on the morphology of the rod-shaped superstructures were investigated. As shown in Fig. 5a, by increasing the tilt angle of the substrate to 3°, the length of the rod-shaped superstructures dramatically reduced to 1.8 μm while the width and spacing exhibited slight changes. As shown in Fig. 5b, by decreasing the concentration of the nanoparticle solution to 40 mM, the spacing of the rod-shaped superstructures significantly increased to ~2 μm, and the size distribution of the rod-shaped superstructures also increased. As presented in Fig. 5c, by reducing the magnitude and gradient of the applied magnetic field to 120 Oe and 40 Oe/cm, respectively, a slight shrink of the superstructure size and an increase of the superstructure spacing were observed. Overall, the tilt angle of the substrate mainly affects the length of the superstructure, while the concentration of the nanoparticle solution has great influence on the spacing of the superstructures. The applied field also plays an important role in determining the size and spacing of the superstructures. Besides, all the superstructures obtained under different experimental conditions show good alignment with the applied magnetic field. It is noteworthy that all the experimental conditions should be adjusted and tuned within proper ranges; otherwise, anisotropic superstructures would not form during the magnetic-field-assisted assembly process. This is because nanoparticle self-assembly has nonlinear nature and it is highly sensitive to the experimental conditions, which will affect the growth and arrangement of the assembled structures [30]. Other experimental conditions such as the boiling point and evaporation rate of the dissolving solvent and also the temperature of the substrate will be investigated in our future research.

**Fig. 6:**
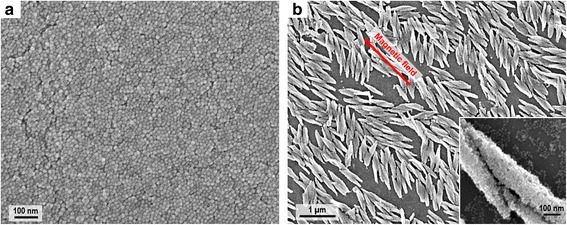
SEM images of the assembled structures prepared with polyhedral IONPs. **a** SEM image of the homogeneous film. **b** SEM image of the spindle-shaped superstructures, with an *inset* of close-up SEM image showing the crystalline texture

Effects of the nanoparticle shape on the morphology and alignment of the superstructures were further investigated. Here, polyhedral IONPs were used instead of spherical IONPs for preparing the sample, and inherently, they could assemble into a closely packed and defect-free film at micron scale as shown in Fig. 6a. Under the in-plane applied magnetic field, spindle-shaped superstructures with a length of 0.8 μm, width of 0.15 μm, and aspect ratio of ~5 were formed as presented in Fig. 6b. The spindle-shaped superstructures have uniform size, but their alignment with the applied field (intersecting angle *β* = 12.9° ± 9.7°) are not as good as the rod-shaped superstructures, and they do not have uniform spacing. The lack of spatial order in these superstructures could be due to the fact that the spindle-shaped superstructures tend to aggregate together, and the shape anisotropy of these superstructures and their constituent polyhedral IONPs should also be taken into consideration. These spindle-shaped superstructures observed here are similar to the loosely aligned micro-rod superstructures reported by Li et al. [23]. The structural difference between these spindle-shaped superstructures and the previously mentioned rod-shaped superstructures could be mainly attributed to the different shapes of their constituent IONPs, which influence the packing and ordering of nanoparticles during self-assembly. Moreover, nanoparticle compositions (related to magnetic properties) and nanoparticle surfactants (affecting diffusion and interaction of nanoparticles in organic solvent) also affect the assembly process and hence the superstructure shape, although their differences are trivial between the two superstructures.

The magnetic-field-assisted assembly of IONPs is a particle deposition process, which depends on the balance between evaporation-driven assembly and field-driven assembly [31, 32]. During the assembly process, convection of the organic solvent due to evaporation drives the IONPs to transport and deposit at the three-phase contact line. The evaporation rate mainly determines the assembly rate, which is quite fast due to that chloroform used for dispersing the IONPs is a highly volatile solvent. The evaporation direction relates to the translation direction of the contact line [33], which determines the assembly direction, and the applied field direction was experimentally adjusted to be parallel to this assembly direction. At the first stage of the assembly process, a gradient magnetic field (typical values magnitude 200 Oe, gradient 60 Oe/cm) was applied in plane, and the dominant forces exerted on IONPs are capillary force induced by solvent evaporation and magnetic force caused by external field and dipolar interactions, while effects of the thermal fluctuations and the local interactions (such as van der Waals attraction and steric repulsion) are negligible [15, 21]. Under the magnetic field, each IONP behaves as a magnetic dipole with magnetic moment *m* = *μ*_0_*MV*, where *μ*_0_ is the permeability of free space and *M* and *V* are the domain magnetization and volume of the IONP, respectively [32]. Magnetic dipole-dipole interaction between two magnetically aligned IONPs is given by *F*_dip_ = 3*m*^2^(1 − 3 cos^2^*α*)/*d*^4^, where *α* is the angle between their magnetic moment and the line connecting their centers and *d* is the inter-particle distance [25, 34]. This interaction is attractive along the field direction but repulsive perpendicular to the field direction [34], which results in the anisotropic growth of spaced superstructures with preferred alignment along the field direction. The gradient of the magnetic field also induces a force along the field direction *F*_mag_ = ∇(*m* ⋅ *B*), with *B* being the magnetic field, and this force together with the capillary force expedites the packing of the IONPs and causes the formation of micron-sized structures. After the evaporation of chloroform (~80 s), assembled structures with uniform size started to develop. At the second stage of the assembly process, a strong and uniform magnetic field with a magnitude of 2 kOe was applied in plane. The IONPs closely packed into condensed superstructures, and their magnetic moments may also get aligned with this strong field. After complete dewetting of the nanoparticle solution (~120 s), the assembled structures became solid coatings on Si. The two stages of the assembly process both affect the morphology and property of the superstructures. Specifically, applying the first stage alone led to micron-sized superstructures without significantly enhanced magnetism, while solely implementing the second stage produced parallel strip patterns with a long length (up to 100 μm) and large spacing (~2 μm).

**Fig. 7:**
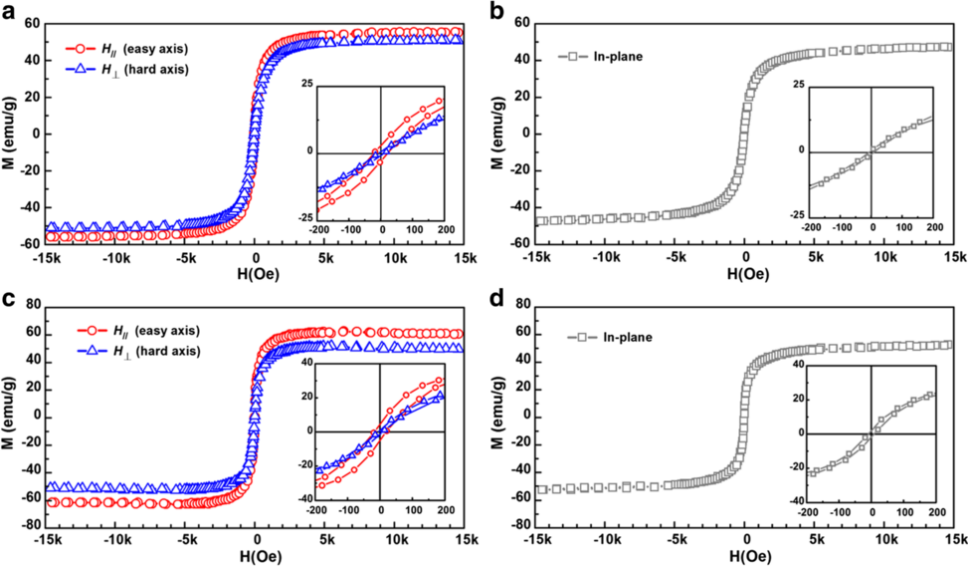
M-H curves of the assembled structures at 300 K. **a** Rod-shaped superstructures of spherical IONPs assembled under a magnetic field. **b** Uniform films of spherical IONPs assembled without a magnetic field. **c** Spindle-shaped superstructures of polyhedral IONPs assembled under a magnetic field. **d** Uniform films of polyhedral IONPs assembled without a magnetic field. *Insets* show the enlarged curves

To probe the magnetic properties of the superstructures, hysteresis loops for the rod-shaped superstructures (Fig. 7a) and spindle-shaped superstructures (Fig. 7c) were measured at 300 K along two in-plane directions, namely, the direction along the applied field (*H*_//_) and the direction perpendicular to the applied field (*H*_⊥_). As a comparison, in-plane hysteresis loops for the spherical IONPs (Fig. 7b) and polyhedral IONPs (Fig. 7d) assembled as uniform films in the absence of a magnetic field were measured, respectively. For the rod-shaped superstructures, coercivity *H*_c_ of 22 Oe and saturation magnetization *M*_s_ of 56 emu/g were observed along *H*_//_, which are larger than the results obtained along *H*_⊥_ (*H*_c_ = 8 Oe, *M*_s_ = 51 emu/g). For the spindle-shaped superstructures, increased *H*_c_ of 18 Oe and increased *M*_s_ of 62 emu/g were also obtained along *H*_//_ compared to the results measured along *H*_⊥_ (*H*_c_ = 6 Oe, *M*_s_ = 52 emu/g). The results indicate that the magnetic easy axes of both superstructures are along the applied field direction and the superstructures show larger coercivity and higher saturation magnetization at their easy axes compared to the uniform films assembled from the same IONPs.

**Fig. 8:**
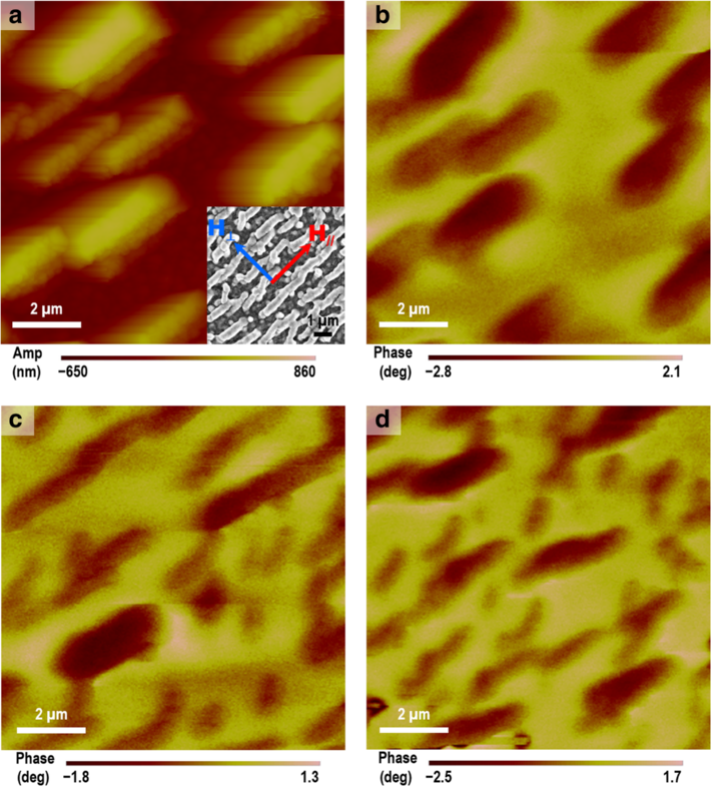
MFM characterization of the rod-shaped superstructures. **a** AFM image of the superstructures, with an *inset* of SEM image. **b** MFM image of the superstructures in a demagnetized state. **c** MFM image of the superstructures in a remanent state after saturation along *H*_//_. **d** MFM image of the superstructures in a remanent state after saturation along *H*_⊥_

To gain insights into the magnetic interactions of the superstructures, MFM imaging was performed for the sample of the rod-shaped superstructures in different magnetization states. The measurements were performed in tapping mode using a magnetized tip (Magnetic Multi75-G, BudgetSensors) through a two-pass process. In the first pass, AFM image of surface topography (Fig. 8a) was recorded, and further analysis of the topographical data indicates a thickness of 0.68 μm for the rod-shaped superstructures, close to the measured width of 0.5 μm from the corresponding SEM image (inset of Fig. 8a). In the second pass, the tip is lifted above the sample surface and scanned at a constant height of 400 nm following the acquired AFM topography. Magnetic contrast of the superstructures was observed on the demagnetized sample by comparing the MFM image (Fig. 8b) with the AFM topography (Fig. 8a) at the same region. This magnetic contrast is probably caused by the MFM tip magnetizing the originally demagnetized sample. It is well known that a MFM tip, being a source of stray field itself, could cause magnetization to the sample it is scanning. Particularly, the building material of our sample is magnetically soft iron oxide nanoparticles with a small coercivity (<25 Oe) and small size (<20 nm), which show nearly superparamagnetic behavior at room temperature; thus, the nanoparticles could be easily magnetized by the stray field of the MFM tip, and the nanoparticle dipole coupling within each superstructure led to the phase difference of the MFM cantilever oscillation [35]. The sample-tip interaction was also reported by Confalonieri et al. in their magnetic system containing a monolayer of 20-nm Fe_2_O_3_ nanoparticles [36]. MFM images of the sample in a remanent state after saturation along *H*_//_ (easy axis) and after saturation along *H*_⊥_ (hard axis) are shown in Fig. 8c, d, respectively. In all the three MFM images (Fig. 8b–d), the superstructures exhibit the same magnetic contrast, indicating that the neighboring superstructures are magnetically coupled together [35]. However, it is not easy to identify the difference between Fig. 8c and d because their magnetic contrasts are similar and also, these two images cannot be directly compared since they are not from exactly the same region. Therefore, the VSM results are more useful and convincing to reflect the magnetism of the superstructures at the easy axis and at the hard axis.

To quantitatively interpret the origin of the enhanced magnetism observed in the VSM results, magnetic anisotropy energy and dipole interaction energy are calculated for the two types of IONPs, respectively. For single-domain non-interacting magnetic nanoparticles, the effective magnetic anisotropy energy constant *K*_eff_ can be calculated as *K*_eff_ = 25*k*_B_*T*_B_/*V*_p_, where *k*_B_ is Boltzmann constant, *T*_B_ is the blocking temperature of nanoparticles, *V*_p_ is the volume of a nanoparticle (${V}_{\mathrm{p}}\approx 1.33\pi {d}_{\mathrm{p}}^3/8$, *d*_p_ is nanoparticle diameter), and *K*_eff_ is the sum of several terms including magnetocrystalline anisotropy, shape anisotropy, surface anisotropy, and stress anisotropy [37]. The calculated *K*_eff_ (2.16 × 10^5^ erg/cm^3^ for 19-nm spherical maghemite IONP; 3.70 × 10^5^ erg/cm^3^ for 16-nm polyhedral magnetite IONP) using *T*_B_ of the nanocrystals in powder form with similar size and shape as our IONPs (225 K; 230 K) [37–39] is much larger than the reported values for bulk maghemite (0.46 × 10^5^ erg/cm^3^) and magnetite (1.35 × 10^5^ erg/cm^3^). This enhancement of magnetic anisotropy is associated with dipole interactions between IONPs. According to the Stoner-Wohlfarth theory, magnetocrystalline anisotropy energy *E*_a_ of a single-domain magnetic nanoparticle is approximated by *E*_a_ = *K*_1_*V*_p_ sin^2^*θ*, where *K*_1_ is the magnetocrystalline anisotropy constant (*K*_1_ ≈ *K*_eff_/12) and *θ* is the angle between the easy axis of the nanoparticle and the direction of the field-induced magnetization [38]. The estimated *E*_a_ is 1.62 × 10^−16^ erg for a spherical IONP using *K*_1_ = 1.8 × 10^4^ emu/cm^3^ and sin *θ* = 0.05, while *E*_a_ is 7.64 × 10^−15^ erg for a polyhedral IONP using *K*_1_ = 3.1 × 10^4^ emu/cm^3^ and sin *θ* = 0.34. Similarly, shape anisotropy energy *E*_sh_ can be approximated as *E*_sh_ = *K*_sh_*V*_p_ sin^2^*θ*, where *K*_sh_ is $0.5{M}_{\mathrm{s}\left(\mathrm{easy}\right)}^2\times \left({N}_{\mathrm{b}}-{N}_{\mathrm{a}}\right)$, with *N*_a_ and *N*_b_ being the demagnetization factors of the nanoparticle in the short-axis and long-axis directions and *M*_s(easy)_ being the saturation magnetization at the easy axis [40, 41]. The estimated *E*_sh_ is 1.71 × 10^−17^ erg for a spherical IONP using *N*_b_ − *N*_a_ = 0.05, *M*_s(easy)_ = 56 emu/g = 274.4 emu/cm^3^, and *K*_sh_ = 1.9 × 10^3^ emu/cm^3^, while *E*_sh_ is 3.72 × 10^−15^ erg for a polyhedral IONP using *N*_b_ − *N*_a_ = 0.3, *M*_s(easy)_ = 62 emu/g = 316.2 emu/cm^3^, and *K*_sh_ = 1.5 × 10^4^ emu/cm^3^. Besides, surface anisotropy also has significant contribution to the effective magnetic anisotropy due to surface spin disorder, while stress anisotropy is much less pronounced considering that the lattice constants of the IONPs are nearly equal to their bulk counterparts [40, 41]. For the assembled superstructure composed of magnetically aligned nanoparticles under the applied magnetic field, the dipole interaction energy of one nanoparticle in the superstructure can be approximated as ${E}_{\mathrm{dip}}=2{\mu}_0{\mu}_{\mathrm{p}}^2/4\pi {r}^3$ using the macro-spin approximation, where *μ*_p_ is the dipole moment of the nanoparticle and *r* is the mean center-to-center distance between the neighboring nanoparticles [42]. By measuring the average inter-particle distance from the SEM images (22 nm for spherical IONP; 18 nm for polyhedral IONP) and assuming *μ*_p_ = *M*_s(easy)_*V*_p_ in a remanent state along the easy axis, the estimated *E*_dip_ is 1.45 × 10^−14^ erg for a spherical IONP, and *E*_dip_ is 1.25 × 10^−14^ erg for a polyhedral IONP. Note that accurate calculation of total dipole interactions in a superstructure is not trivial, due to their long-range effects, highly anisotropic nature, and their dependence on the arrangement of the constituent nanoparticles and the direction of nanoparticle dipole moments [43]. Here, the estimated dipole interaction energy is comparable to the estimated magnetic anisotropy energy for both types of IONPs, indicating that it is also of great importance in determining the magnetic properties of the interacting nanoparticles. Furthermore, by modeling the superstructures as aligned arrays of nanowires, the corresponding coercivity along the easy axis can be evaluated as ${H}_{\mathrm{c}\left(\mathrm{easy}\right)}=4{L}_{\mathrm{ex}}^2{q}^2{M}_{\mathrm{s}\left(\mathrm{easy}\right)}/{D}_{\mathrm{w}}^2+{P}_c{K}_1^2{d}_{\mathrm{p}}^2/{M}_{\mathrm{s}\left(\mathrm{easy}\right)}{A}_{\mathrm{ex}}$, where the first term is related to the shape anisotropy energy of nanowires, the second term is related to the magnetocrystalline anisotropy energy of nanoparticles [35, 42, 44], *L*_ex_ is the exchange length, *q* is the smallest solution of the Bessel function relating to the aspect ratio (1.84), *D*_w_ is the average diameter of nanowires, *P*_c_ is a coefficient (0.5), and *A*_ex_ is the exchange stiffness constant (1 × 10^−6^ erg/cm). The estimated *H*_c(easy)_ is 6.6 Oe for the rod-shaped superstructures using *L*_ex_ = 5 nm and *D*_w_ = 500 nm, while *H*_c(easy)_ is 11 Oe for the spindle-shaped superstructures using *L*_ex_ = 2.4 nm and *D*_w_ = 208 nm. Both of the calculated *H*_c(easy)_ values are smaller than the experimentally obtained *H*_c_ values (22 Oe; 18 Oe), due to that the estimation of coercivity using the nanowire model does not consider the magnetic interactions between different superstructures and the dipole interactions between different nanoparticles [35]. Above all, the enhanced magnetism in the superstructures is mainly attributed to the magnetic anisotropy of the constituent nanoparticles, the strong dipole interactions between the neighboring nanoparticles within each superstructure, and the increased shape anisotropy of the superstructures. Additionally, the building blocks of the superstructures are magnetically soft IONPs with sub-20-nm sizes, which show nearly superparamagnetic behavior at room temperature. If other magnetic nanoparticles with strong ferromagnetism (for example, CoFe_2_O_4_) [45] are used for the magnetically directed assembly, much more significant enhancement of coercivity and saturation magnetization in the resulting superstructures could be anticipated. Future works will be carried out to fabricate anisotropic superstructures using magnetically hard nanoparticles (sub-20-nm cobalt ferrite nanoparticles) and also develop uniform superstructures with controllable size (from 2 μm down to 200 nm) and tunable magnetic properties (including strength of magnetic coercivity and orientation of magnetic easy axis) for the application of high-density magnetic storage devices and magnetic sensors.

## Conclusions

In summary, sub-20-nm IONPs with spherical shape and polyhedral shape were chemically synthesized, and self-assembly of these IONPs into anisotropic superstructures under the in-plane magnetic field was demonstrated. The solvent-evaporation assembly process expedited by an external magnetic field produced nanoparticle superstructures at sub-10-μm sizes with homogeneous morphology and controlled spacing. The experimental conditions exhibit influence on the size and spacing of the superstructures, while the shape of the building nanoparticles affects the morphology and alignment of the superstructures. Well-aligned rod-shaped superstructures were obtained using spherical IONPs, and loosely aligned spindle-shaped superstructures were prepared with polyhedral IONPs. The two types of superstructures both show magnetic easy axes along the applied field direction, and they also show enhancement of coercivity and saturation magnetization along their easy axes. The enhanced magnetism in the superstructures is associated with magnetic anisotropy and dipole interactions of the nanoparticles and shape anisotropy of the superstructures. This low-cost technique of magnetically directed nanoparticle assembly could be used for fabricating nanomaterial-based structures with controlled geometric dimensions and enhanced magnetic properties for magnetic and energy storage applications. The anisotropic superstructures prepared here are promising for studying fundamental physics related to magnetic interactions and collective behaviors of magnetic nanomaterials.
